# Pharmacokinetic Comparisons of Multiple Triterpenic Acids from *Jujubae Fructus* Extract Following Oral Delivery in Normal and Acute Liver Injury Rats

**DOI:** 10.3390/ijms19072047

**Published:** 2018-07-13

**Authors:** Yao Li, Sheng Guo, Quanjin Ren, Dandan Wei, Ming Zhao, Shulan Su, Zhishu Tang, Jin-Ao Duan

**Affiliations:** 1 Jiangsu Collaborative Innovation Center of Chinese Medicinal Resources Industrialization/State Key Laboratory Cultivation Base for Traditional Chinese Medicine Quality and Efficacy, Nanjing University of Chinese Medicine, Nanjing 210023, China; liyaonjutcm@163.com (Y.L.); wei.dandan@njucm.edu.cn (D.W.); mingzhao@njucm.edu.cn (M.Z.); sushulan@njucm.edu.cn (S.S.); 2 Institute of Botany, Jiangsu Province and Chinese Academy of Science, Nanjing 210014, China; renquanjin@cnbg.net; 3 Shaanxi Collaborative Innovation Center of Chinese Medicinal Resources Industrialization, Shaanxi University of Chinese Medicine, Xianyang 712046, China; tzs6565@163.com

**Keywords:** *Ziziphus jujuba*, triterpenic acids, pharmacokinetic study, acute liver injury

## Abstract

*Jujubae Fructus*, the dried fruit of *Ziziphus jujuba*, has been used as Chinese medicine and food for centuries. Triterpenic acids have been found to be the major bioactive constituents in *Jujubae Fructus* responsible for their hepatoprotective activity in previous phytochemical and biological studies, while few pharmacokinetic studies have been conducted. To reveal the kinetics of the triterpenic acids under the pathological liver injury state, an established ultra-performance liquid chromatography coupled with a mass spectrometry method was applied for the simultaneous quantitation of seven triterpenic acids (ceanothic acid, epiceanothic acid, pomonic acid, alphitolic acid, maslinic acid, betulinic acid, and betulonic acid) in plasma samples of normal and acute liver injury rats induced by CCl_4_. The results showed that there were significant differences (*p* < 0.05) in the pharmacokinetic parameters of seven triterpenic acids between model and normal groups. The AUC_0–t_ and AUC_0–∞_ of epiceanothic acid (5227 ± 334 μg⋅h/L vs. 1478 ± 255 μg⋅h/L and 6127 ± 423 μg⋅h/L vs. 1482 ± 255 μg⋅h/L, respectively) and pomonic acid (4654 ± 349 μg⋅h/L vs. 1834 ± 225 μg⋅h/L and 4776 ± 322 μg⋅h/L vs. 1859 ± 230 μg⋅h/L, respectively) in model rats were significantly higher than those in normal rats, and the CLz/F of them were significantly decreased (0.28 ± 0.02 L/h/kg vs. 1.36 ± 0.18 L/h/kg and 19.96 ± 1.30 L/h/kg vs. 53.15 ± 5.60 L/h/kg, respectively). In contrast, the above parameters for alphitolic acid, betulinic acid and betulonic acid exhibited the quite different trend. This pharmacokinetic research might provide useful information for the clinical usage of triterpenic acids from *Jujubae Fructus*.

## 1. Introduction

*Jujubae Fructus*, the fruit of *Ziziphus jujuba* Mill., has been used as herb medicine and food for centuries in China [1,2]. According to traditional Chinese medicine theory, *Jujubae Fructus* could reinforce spleen and stomach, and is commonly used for the treatment of anorexia, fatigue and loose stools related to deficiency syndromes of the spleen and hysteria in women [3]. The controlled clinical trials also showed that *Jujubae Fructus* extract is an effective treatment for chronic constipation [4] and type 2 diabetes [5]. In addition, in northern China, the decoction of *Jujubae Fructus* are claimed as useful remedies for the management and/or control of hepatitis in folk [6].

In support of its traditional efficacy, modern researches have revealed that *Jujubae Fructus* has pharmacological properties including hepatoprotective [6], gastrointestinal protective [7], anti-inflammatory [8], immunomodulating [9] and hematopoiesis effects [10]. Among them, ethanolic extract of *Jujubae Fructus* with a dose of 200 mg/kg could significantly decrease ALT and AST, and attenuate histopathology of hepatic injury induced by carbon tetrachloride (CCl_4_), and the results indicated that hepatic protective effects of *Jujubae Fructus* were relevant to modulate the oxidative stress in hepatic injury [6,11]. These results further confirmed the traditional efficacy of *Jujubae Fructus* on the hepatoprotective effect. Phytochemical and biological studies showed that these multiple bioactivities of *Jujubae Fructus* could be attributed to its various constituents, such as triterpenic acids [12], polysaccharides [2], phenolic acids [13], flavonoids [14], nucleoside and amino acids [15,16]. Among these components, triterpenic acids, such as betulinic acid, alphitolic acid, maslinic acid, etc., have been reported to possess biological effects of hepatoprotective, anti-inflammatory, antimicrobial and antioxidant activities [6,8,17,18], which have attracted great attention from researchers.

The liver is a crucial organ for metabolism and detoxification in the human body, and liver disease has nowadays become one of the most common causes of death [19]. There are reports that acute liver injury is the main initiating factor and pathological basis for liver fibrosis, hepatitis, cirrhosis and even liver cancer, which could result in terminal liver failure [20,21]. The previous studies have reported the hepatoprotective effect of *Jujubae Fructus*, and triterpenic acids have been considered as the main bioactive compounds for the above activity [6,11]. However, there were few reports on the pharmacokinetic studies of triterpenic acids from *Jujubae Fructus* in liver injury model animals, and relevant pharmacokinetic studies of *Jujubae Fructus* in humans were also rare. It is well known that under the pathological condition of liver injury, pharmacokinetic and metabolic behaviors of drugs are often altered [20,22,23]. Thus, based on the ultra-performance liquid chromatography coupled with mass spectrometry (UHPLC-MS/MS) method established in the previous study [24], the pharmacokinetics of triterpenic acids from *Jujubae Fructus* in normal and acute liver injury rats were compared in this paper for the purpose of providing clinical reference for *Jujubae Fructus*.

## 2. Results and Discussion

### 2.1. Validation of the Acute Liver Injury Rats Model

**Figure 1:**
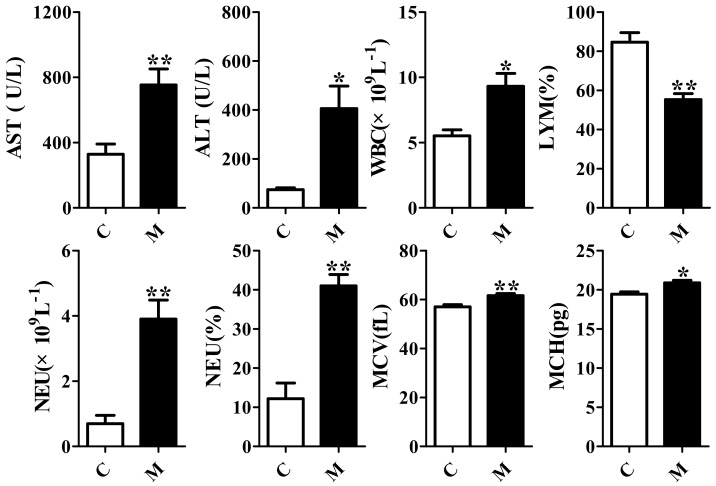
Changes in alanine aminotransferase (ALT), aspartate aminotransferase (AST), and peripheral blood routine between control group (C) and model group of acute liver injury (M). WBC: white blood cell count, LYM: lymphocyte ratio, NEU: neutrophil count/ratio, MCV: erythrocyte mean corpuscular volume, MCH: mean corpuscular hemoglobin (means ± SEM, *n* = 6, * *p* < 0.05, ** *p* < 0.01 vs. control group).

**Figure 2:**
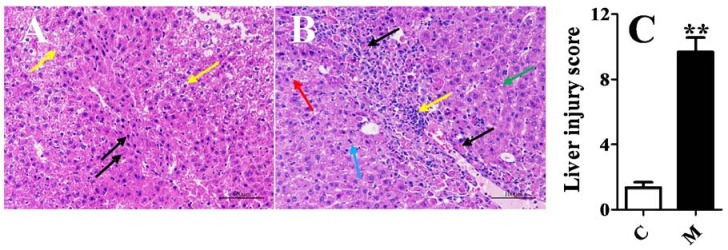
Pathological sections of liver, means ± SEM, *n* = 6, ** *p* < 0.01 vs. control group. (**A**) blank group: Local hepatocellular necrosis, nucleus fragmentation dissolves or pyknosis, and eosinophilic cytoplasm can be seen as shown by **black** arrows; degeneration of hepatocytes, swelling of cell bodies, irregular vacuoles and eosinophilic particles in the cytoplasm were shown by **yellow** arrows; (**B**) acute liver injury group: hepatocyte necrosis, nucleus fragmentation or dissolution, and eosinophilic cytoplasmic enhancement were shown by the **black** arrow; inflammatory cell infiltration was indicated by the **yellow** arrow; hepatic cell vesicle steatosis was shown by the **green** arrow; some hepatocytes in the vicinity of the necrotic lesions are degenerated, as indicated by the **red** arrow; pathological mitoses can be seen, as indicated by the **blue** arrows; and (**C**) Column chart of liver injury score.

To verify whether the rat model of acute liver injury was successful, peripheral blood routine, levels of alanine aminotransferase (ALT), aspartate aminotransferase (AST) and histopathological characteristics were analyzed. All the results are presented in Figure 1 and Figure 2. It was shown that the liver injury score and the levels of white blood cell count (WBC), neutrophil (NEU) count and ratio, erythrocyte mean corpuscular volume (MCV), mean corpuscular hemoglobin (MCH), AST and ALT of the model group after being injected intraperitoneally with CCl_4_ increased significantly (*p* < 0.05) compared to control group. Lymphocyte (LYM) ratios of the model group decreased significantly. It was known that the levels of AST and ALT were all related to liver function. Besides, it has reported that liver injury induced by CCl_4_ could cause inflammation [19,25], which could be presented with the increases of WBC and NEU. Thus, the above results indicated that the acute liver injury rat model was successful, and could be used for the following experiment.

### 2.2. Method Validation

The method for separation and detection of analytes was performed in the established UHPLC-MS/MS method previously [24] with appropriate adjustments.

#### 2.2.1. Selectivity

**Figure 3:**
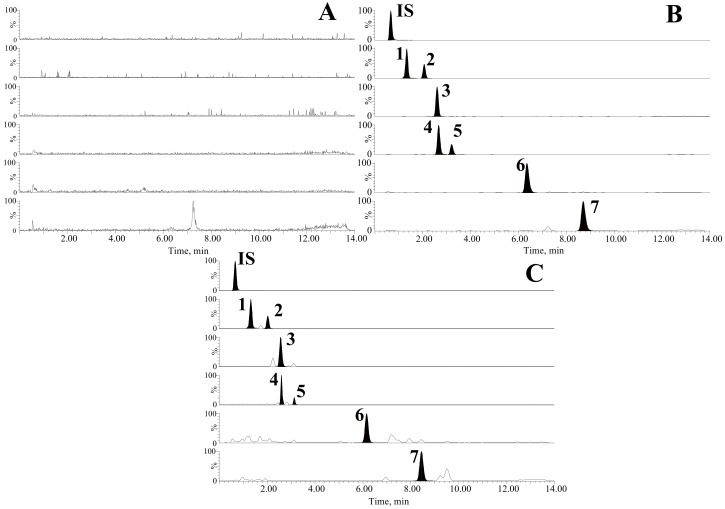
Typical UHPLC-MS/MS chromatograms of (**A**) blank plasma, (**B**) blank plasma spiked with the analytes and IS, and (**C**) plasma sample from a normal rat at 6 h after oral administration of TAE, which were detected with multiple reaction monitoring mode.; ceanothic acid (1), epiceanothic acid (2), pomonic acid (3), alphitolic acid (4), maslinic acid (5), betulinic acid (6), betulonic acid (7).

Figure 3 showed the chromatograms obtained from the blank plasma of a rat, blank plasma spiked with the standards of seven mixed triterpenic acids and internal standard (IS), and rat plasma acquired at 6 h after oral administration of triterpenic acids extract (TAE). No significant endogenous interference or metabolites were found in the blank plasma at the retention times of standards and IS, which revealed that the selectivity of the method was acceptable.

#### 2.2.2. Linearity and Lower Limit of Quantification (LLOQ)

The linearity of the proposed method was evaluated by means of representative calibration curves, correlation coefficients and a linear range of the seven standards, and LLOQs were used for determining the sensitivity of the method. As shown in Table 1, all the correlation coefficients (*R*^2^) are ≥0.9930, which indicated the good linearity of all analytes and the LLOQs of the seven triterpenic acids in plasma were suitable for quantitative detection.

#### 2.2.3. Precision and Accuracy

As shown in Table 2, the deviation in intra- and inter-day precision of all the analytes in QC samples were ≤10.11% and ≤14.29%, respectively, and the accuracies (RE) of those analytes ranged from −2.03% to 14.87%. The results indicated that the method was accurate, precise and was acceptable for analysis of biological samples due to the values being within the acceptable criteria.

#### 2.2.4. Extraction Recovery and Matrix Effect

The results of extraction recovery and the matrix effect are shown in Table 3. It was shown that the extraction recoveries ranged from 78.98% to 103.8%, and the matrix effects were between 75.28% and 109.3% with the RSD values less than 15.0% for the seven analytes at three QC concentrations. As for IS, the extraction recoveries and matrix effects were 87.37–98.45% and 75.52–80.31%, respectively. All the results suggested the reliable extraction recoveries of these analytes and no significant matrix effect in this experiment.

#### 2.2.5. Stability

The QC samples with different conditions (three freeze-thaw cycles; 12 h at room temperature; 24 h at 4 °C; 20 days at −20 °C) were used to investigate the stability of the seven triterpenic acids, and the results (Table 4) showed that the RSD values were all less than 13.59%, which indicated that all analytes were stable throughout the whole test.

### 2.3. Pharmacokinetic Study

**Figure 4:**
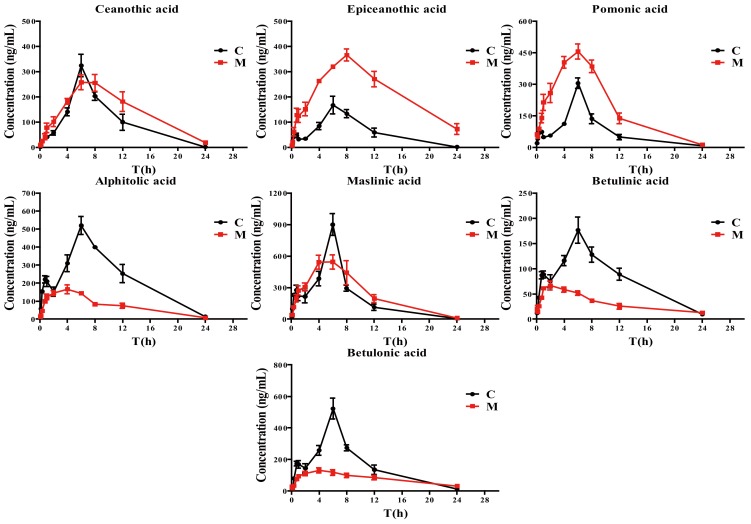
Mean plasma concentration–time curves of seven triterpenic acids after oral administration of TAE for control (C) and acute liver injury model groups (M) (means ± SEM, *n* = 6).

The pharmacokinetics of seven triterpenic acids in plasma after a single oral administration of TAE in normal and acute liver injury rats were analyzed by the validated UHPLC-MS/MS method. The pharmacokinetic parameters obtained with the non-compartment module of Drug and Statistic (DAS) 3.2.8 pharmacokinetic software are listed in Table 5. The mean concentration-time profiles are presented in Figure 4.

As shown in Figure 4, the consistent plasma concentration-time profiles in normal rats were found for these seven analytes, which may be attributed to their similar chemical structures. However, this phenomenon was not found in the acute liver injury model, which could be ascribed to the pathological changes of the liver. Moreover, certain pharmacokinetic parameters for these triterpenic acids in acute liver injury rats showed significant differences from those in normal rats, especially for the area under the time curve (AUC_0–t_ and AUC_0–∞_) and the apparent plasma clearance (CLz/F). The AUC_0–t_ and AUC_0–∞_ of epiceanothic acid and pomonic acid achieved from the drug concentration-time in acute liver injury rats after oral administration of TAE were significantly higher than those in normal rats, and the CLz/F of them were significantly decreased. These indicated that the acute liver injury could increase the bioavailiability of epiceanothic acid and pomonic acid, and decrease their elimination. In contrast, the mean AUC_0–t_, AUC_0–∞_ and the mean peak concentration (*C*_max_) of alphitolic acid, betulinic acid and betulonic acid in acute liver injury rats were achieved with relatively lower values compared to those in normal rats. The obvious higher CLz/Fs of alphitolic acid, betulinic acid and betulonic acid compared to normal rats were also found. The above results suggested that the systemic exposure of alphitolic acid, betulinic acid and betulonic acid were weakened and the elimination increased under the liver injury pathological condition. Additionally, the *T*_max_ of betulinic acid and betulonic acid of the model group were lower than those of the normal group (*p* < 0.05), and the AUC_0–t_ of ceanothic acid and the *C*_max_ of epiceanothic acid were significantly higher in acute liver injury rats. The other pharmacokinetic parameters in the model rats were found to be different but not significant compared with the normal rats.

It is well known that the liver plays important roles in drug biotransformation, metabolism, detoxification and so on [19]. It contains various enzymes involved in drug metabolism, including cytochrome P450 which converts the drug to active metabolites and directly affects the rate of metabolism [26]. Liver injury might lead to liver cell degeneration, necrosis and changes in cytochrome P450 isoenzyme contents, which could alter the disposition of drugs in the body [22,27]. There have been reports on changes in biotransformation, clearance and pharmacokinetics of drugs in liver injury [28].

Besides, the influence of intestinal drug transport and its microbiota might be an important factor in the absorption and bioavailability of the orally administered medicines. It has been reported that liver injury often causes the increase in intestinal permeability and endotoxin, and the disorder of the intestinal microbiota [29,30,31]. The increased endotoxin could further cause liver injury more severely and might be a vicious cycle [30]. All the above reasons might synthetically result in differences in pharmacokinetic behavior between acute liver injury and normal rats after oral administration of TAE. However, the hypotheses are still undefined and need further validation.

It is worth noting that the plasma concentration–time profiles of epiceanothic acid and pomonic acid were markedly different from alphitolic acid, betulinic acid and betulonic acid in acute liver injury rats administered TAE from Jujubae Fructus. The AUC_0–t_ and AUC_0–∞_ of epiceanothic acid and pomonic acid achieved from the drug concentration-time in acute liver injury rats were significantly higher than those in normal rats, and the CLz/F of them were significantly decreased. In contrast, the mean AUC_0–t_, AUC_0–∞_ and CLz/F of alphitolic acid, betulinic acid and betulonic acid in acute liver injury rats showed quite a different trend. This phenomenon might be attributed to their subtle difference in chemical structures which could lead to different metabolic pathways. Therefore, further studies for the investigation of triterpenic acids metabolism and distribution in vivo are warranted.

At present, there are some clinical reports about the liver protection of *Jujubae Fructus*, but there is little relevant pharmacokinetic study of *Jujubae Fructus* in humans. Thus, the depth clinical studies to validate the proposed hypothesis need to be conducted in the future.

## 3. Materials and Methods

### 3.1. Chemicals and Reagents

Acetonitrile and methanol were purchased from Merck KGaA (Darmstadt, Germany). Chloramphenicol used as the internal standard (IS) was obtained from Aladdin reagent Co., Ltd. (Shanghai, China). Ammonium acetate and CCl_4_ were purchased from Sinopharm Chemical Reagent Co., Ltd. (Shanghai, China). Deionized water was prepared by a Milli-Q system (Millipore, Bedford, MA, USA). *Jujubae Fructus* was gathered at Liuling, Shanxi Province, China. The standards (>98% purity) including ceanothic acid, epiceanothic acid, pomonic acid, alphitolic acid, maslinic acid, betulinic acid and betulonic acid were isolated from *Z. jujuba* fruits in our laboratory, and their structures were identified by NMR, HPLC and MS. Other reagents used were of analytical grade.

TAE of *Jujubae Fructus* prepared in our previous experiment [24] was used in this experiment, which contains ceanothic acid, epiceanothic acid, pomonic acid, alphitolic acid, maslinic acid, betulinic acid, and betulonic acid with the contents of 0.78, 0.44, 24.08, 4.97, 17.31, 16.79 and 14.29 mg/g, respectively.

### 3.2. Instrumentation and Chromatographic Conditions

A Waters Acquity™ UPLC system (Waters Corp., Milford, MA, USA) equipped with a Waters Xevo™ TQ/MS (Waters Corp.) was used. Separation and detection of analytes was performed with the established method described previously [24]. Data acquired was analyzed by MassLynx V4.1 workstation (Waters Corp.).

### 3.3. Animals and Induction of Acute Liver Injury

Male Sprague-Dawley rats (SPF, 220-240 g) were bought from Experimental Animal Center of Zhejiang Province and the permit number was SCXK (zhe) 2014-0001 (project identification code: No1703300021, 31/03/2017). Animals were housed in Drug Safety Evaluation Center of Nanjing University of Chinese Medicine, Nanjing, China. Animal welfare and all experimental protocols were performed in accordance with the Regulations of Experimental Animal Administration (State Committee of Science and Technology of the People’s Republic of China) and approved by the Animal Ethics Committee of Nanjing University of Chinese Medicine. These rats were housed under standard environment with food and water provided ad libitum. After adaptation for 7 days, the 12 rats were randomly divided into 2 groups: control group (C) and acute liver injury group (model group, M). Rats of M group were injected intraperitoneally with 50% CCl_4_ once at a dose of 2 mL/kg. CCl_4_ used for injection was dissolved in peanut oil. To verify whether the model was successful, whole blood and serum samples were collected from the retro-orbital plexus of rats after modeling for measuring the peripheral blood routine parameters using ADVIA120 fully automatic blood analyzer (Bayer, Germany) and the levels of ALT and AST with Dimension Xpand automatic biochemical analyzer (Bayer, Germany). Furthermore, after the final collection of blood, the rats were anesthetized and sacrificed with 10% choral hydrate (350 mg/kg *ip*) and the liver samples were taken and fixed in formaldehyde solution for histopathological analysis.

### 3.4. Sample Preparation

After being thawed at room temperature (18–25 °C), each plasma sample (100 μL) was precipitated with 300 μL acetonitrile and 20 μL IS. The mixture was vortexed for 1 min and centrifuged at 15,000× *g* for 15 min. Then, 2 μL of supernatant was injected for UHPLC-MS/MS analysis.

### 3.5. Preparation of Standard Solutions, Calibration Standards and Quality Control (QC) Samples

A mixed stock solution containing 126.9 μg/mL of ceanothic acid, 129.4 μg/mL of epiceanothic acid, 125.6 μg/mL of pomonic acid, 124.4 μg/mL of alphitolic acid, 126.3 μg/mL of maslinic acid, 130.0 μg/mL of betulinic acid and 129.4 μg/mL of betulonic acid was prepared with methanol as a solvent. A series of working standard solutions were prepared from the mixed stock solution by sequential dilution with methanol. Calibration solutions were prepared by spiking 10 μL of working solution into 100 μL blank plasma to obtain a mixed final dilution of 2.31–2951 ng/mL ceanothic acid, 2.35–3009 ng/mL epiceanothic acid, 2.28–2922 ng/mL pomonic acid, 2.26–2892 ng/mL alphitolic acid, 2.29–2936 ng/mL maslinic acid, 2.36–3023 ng/mL betulinic acid, and 2.35–3009 ng/mL betulonic acid. The quality control (QC) samples were prepared at low, medium, and high concentrations (23.05, 368.8, and 2951 ng/mL ceanothic acid, 23.51, 376.1, and 3009 ng/mL epiceanothic acid, 22.82, 365.2, and 2922 ng/mL pomonic acid, 22.60, 361.6, and 2892 ng/mL alphitolic acid, 22.94, 367.0, and 2936 ng/mL maslinic acid, 23.62, 377.9, and 3023 ng/mL betulinic acid, and 23.51, 376.1, and 3009 ng/mL betulonic acid) in the same way as calibration solutions. The stock solution of IS (9.76 μg/mL chloramphenicol) was also prepared in methanol.

### 3.6. Method Validation

This proposed method was validated according to US-FDA Bioanalytical Method Validation Guidance [32], and selectivity, linearity, precision, extraction recovery, matrix effect and stability were assessed.

#### 3.6.1. Selectivity

The chromatograms of the rat blank plasma, blank plasma spiked with the seven standards and IS, and rat plasma acquired at 6 h after gavage of TAE, were analyzed and compared to investigate the selectivity of the method [33].

#### 3.6.2. Linearity and LLOQ

The peak area ratio (*y*) of the analyte to the IS vs. the nominal concentration (*x*, ng/mL) was used to plot the calibration curve and determine the linearity with weighted (1/*x*^2^) least square linear regression [34]. LLOQ of the method was determined based on the signal to noise ratio of 10:1 with the acceptable precision in six replicates of blank plasma (RSD ≤ 20%) [35].

#### 3.6.3. Accuracy and Precision

The intra-day and inter-day accuracy and precision were assessed by determining the concentration of six replicates of QC samples (as described in section ‘3.5’) at three concentration levels (low, medium and high) on the same day and on three consecutive days, respectively. The accuracy was described as relative error (RE, %) and precision was expressed as relative standard deviation (RSD, %) [36]. The acceptability criteria for accuracy and precision were required within ±15% according to the guidelines of FDA.

#### 3.6.4. Recovery and Matrix Effect

Extraction recovery and matrix effect were evaluated with six replicates of QC samples at three concentrations. Extraction recovery of the seven triterpenic acids were performed by comparing the peak area of every analyte extracted from plasma samples with that of post-extraction spiked plasma blank [24]. For evaluation of the matrix effect, the peak areas of the analytes in post-extraction standard plasma samples (B) were compared with those of pure methanol containing an equivalent amount of standards at QC levels (A) [37].

#### 3.6.5. Stability

The stability of analytes in rat plasma were assessed by analyzing six replicates of QC samples at three concentration levels. The QC samples in different storage conditions including three freeze-thaw cycles (from −80 °C to room temperature), 12 h at room temperature, and 20 days at −20 °C, were used to evaluate the freeze-thaw, short-term, and long-term stability, respectively. In addition, the autosampler stability was also evaluated after samples were stored in the autosampler at 4 °C for 24 h [38].

### 3.7. Pharmacokinetic Study in Rat and Statistical Analysis

After fasting for 12 h with free access to water, rats in both C and M groups were intragastrically administered TAE (dissolved in water with 10% tween-80) at a dose of 4.0 g/kg. The administered volume was 10 mL/kg for each rat, each time. Serial blood samples (about 500 μL for each) were collected into heparinized tubes from the retro-orbital plexus at 0, 5, 10, 20, 45, 60, 120, 240, 360, 480, 720 and 1440 min after oral administration. All blood samples were centrifuged at 3500 rpm at 4 °C for 10 min, then the supernatants were separated and stored at −80 °C until analysis. The pharmacokinetic parameters were calculated by Drug and Statistic (DAS) 3.2.8 pharmacokinetic software in a non-compartment model. The experimental data were expressed as mean ± SEM. Independent Samples *t*-test via the software SPSS 22.0 (IBM SPSS, Chicago, IL, USA) was used for evaluation of statistical significance.

## 4. Conclusions

A rapid, sensitive, and simple UHPLC-MS/MS method was used for the determination of seven triterpenic acids in the plasma of acute liver injury and normal rats after oral administration of TAE. The results demonstrated that acute liver injury induced by CCl_4_ could alter the pharmacokinetic parameters of seven triterpenic acids, such as AUC_0–t_, AUC_0–∞_, CLz/F and C_max_. And the differences might be due to the changes in liver function, intestinal permeability and intestinal microbiome in the acute liver injury pathological state. These pharmacokinetic results in the pathological state of acute liver injury might provide more useful information for the application of *Jujubae Fructus* in treating liver disease.

## Figures and Tables

**Table 1:** Regression equation and LLOQ of seven compounds.

Compound	Linear Regression Equation	*R* ^2^	Range (ng/mL)	LLOQ (ng/mL)
Ceanothic acid	*y* = 2.347 × 10^−3^ *x* + 8.488 × 10^−2^	0.9982	4.61–2951	2.93
Epiceanothic acid	*y* = 1.832 × 10^−3^ *x* − 3.799 × 10^−2^	0.9987	2.35–3009	0.92
Pomonic acid	*y* = 2.989 × 10^−4^ *x* − 1.377 × 10^−3^	0.9997	22.82–2922	6.47
Alphitolic acid	*y* = 4.411 × 10^−3^ *x* + 4.185 × 10^−1^	0.9930	22.60–2892	7.34
Maslinic acid	*y* = 1.657 × 10^−3^ *x* + 1.111 × 10^−1^	0.9968	22.94–2936	15.02
Betulinic acid	*y* = 6.084 × 10^−3^ *x* + 9.910 × 10^−2^	0.9996	23.62–3023	17.23
Betulonic acid	*y* = 3.058 × 10^−3^ *x* + 1.718 × 10^−2^	0.9999	23.51–3009	22.68

**Table 2:** Precision and accuracy for the determination of the seven compounds.

Compound	Concentration (ng/mL)	Intra-Day		Inter-Day	
		Accuracy (RE, %)	Precision (RSD, %)	Accuracy (RE, %)	Precision (RSD, %)
Ceanothic acid	23.05	14.39	1.98	9.85	6.29
	368.8	14.12	1.68	8.64	7.18
	2951	5.62	8.49	4.77	8.03
Epiceanothic acid	23.51	10.40	3.94	5.66	11.33
	376.1	9.97	1.33	5.95	5.43
	3009	5.04	7.30	2.74	6.52
Pomonic acid	22.82	−2.03	10.11	9.02	9.31
	365.2	13.73	6.63	11.33	12.443
	2922	9.42	9.49	11.09	9.75
Alphitolic acid	22.60	12.42	1.38	10.95	13.39
	361.6	12.18	2.39	9.76	5.14
	2892	5.87	7.69	1.19	5.90
Maslinic acid	22.94	12.03	4.88	14.61	14.29
	367.0	14.87	1.17	12.99	9.18
	2936	12.44	6.58	8.21	5.77
Betulinic acid	23.62	12.86	7.05	11.93	8.80
	377.9	13.93	3.97	10.59	7.69
	3023	4.42	5.63	2.43	4.96
Betulonic acid	23.51	13.50	7.82	10.31	12.88
	376.1	12.88	2.14	10.52	8.78
	3009	8.41	8.57	5.52	8.75

**Table 3:** Recoveries and matrix effects of the seven compounds in rat plasma.

Compound	Concentration (ng/mL)	Recovery (%, Mean ± S.D.)	Matrix Effect (%, Mean ± S.D.)
Ceanothic acid	23.05	87.77 ± 3.88	94.42 ± 4.37
	368.8	89.99 ± 6.08	91.57 ± 12.23
	2951	83.88 ± 2.30	82.64 ± 1.67
Epiceanothic acid	23.51	91.06 ± 13.06	91.71 ± 8.25
	376.1	90.87 ± 6.21	108.4 ± 15.8
	3009	82.01 ± 1.28	105.9 ± 1.6
Pomonic acid	22.82	78.98 ± 2.93	75.28 ± 9.39
	365.2	89.32 ± 5.73	94.82 ± 12.17
	2922	83.31 ± 3.72	109.3 ± 3.9
Alphitolic acid	22.60	90.09 ± 3.62	85.95 ± 6.71
	361.6	93.48 ± 5.93	82.60 ± 7.93
	2892	85.33 ± 0.80	77.22 ± 0.67
Maslinic acid	22.94	94.67 ± 8.49	89.02 ± 8.68
	367.0	93.23 ± 5.66	93.72 ± 8.65
	2936	83.58 ± 2.24	89.37 ± 1.33
Betulinic acid	23.62	103.8 ± 11.3	89.83 ± 10.73
	377.9	100.5 ± 8.9	101.8 ± 14.8
	3023	89.34 ± 1.81	91.10 ± 0.28
Betulonic acid	23.51	101.0 ± 8.1	92.62 ± 7.51
	376.1	101.7 ± 7.2	100.4 ± 13.5
	3009	85.79 ± 2.10	102.0 ± 2.0

**Table 4:** Stabilities of the seven compounds in rat plasma.

Compound	Concentration (ng/mL)	Three Freeze-Thaw Cycles (RSD%)	12 h at Room Temperature (RSD%)	24 h at 4 °C (RSD%)	20 Days at −20 °C (RSD%)
Ceanothic acid	23.05	9.10	12.59	10.45	8.57
	368.8	2.56	10.12	5.91	2.04
	2951	3.41	11.68	8.14	7.15
Epiceanothic acid	23.51	9.22	13.33	9.92	6.33
	376.1	3.05	9.67	6.30	1.65
	3009	5.46	12.27	8.07	6.30
Pomonic acid	22.82	13.42	13.59	12.50	11.71
	365.2	5.33	10.54	7.15	7.01
	2922	4.17	12.67	8.42	7.72
Alphitolic acid	22.60	9.78	11.32	11.99	1.82
	361.6	3.06	8.64	6.14	2.88
	2892	4.28	11.39	9.52	4.90
Maslinic acid	22.94	10.21	9.47	13.61	10.65
	367.0	3.10	7.76	5.34	1.17
	2936	5.54	11.54	9.72	6.31
Betulinic acid	23.62	10.49	11.32	9.31	5.82
	377.9	3.61	6.96	5.46	3.35
	3023	5.32	11.95	8.62	4.50
Betulonic acid	23.51	11.18	10.50	4.53	7.47
	376.1	3.44	6.99	4.08	2.48
	3009	6.47	11.46	6.13	8.51

**Table 5:** Pharmacokinetic parameters of seven compounds after an oral administration in normal and model rats (means ± SEM, *n* = 6).

Compound	Group	*C*_max_	CLz/F	*T*_max_	*T*_1/2z_	AUC_0–t_	AUC_0–∞_
		(μg/L)	(L/h/kg)	(h)	(h)	(μg⋅h/L)	(μg⋅h/L)
Ceanothic acid	C	326.9 ± 67.4	1.25 ± 0.08	6.67 ± 0.47	2.06 ± 0.26	2474 ± 168	2479 ± 171
	M	286.5 ± 21.1	0.87 ± 0.06	8.67 ± 1.25	4.11 ± 0.48	3431 ± 171 *	3567 ± 232
Epiceanothic acid	C	169.7 ± 34.4	1.36 ± 0.18	7.33 ± 0.47	2.45 ± 0.03	1478 ± 255	1482 ± 255
	M	371.9 ± 19.9 *	0.28 ± 0.02 *	7.33 ± 0.47	7.00 ± 1.33	5227 ± 334 **	6127 ± 423 **
Pomonic acid	C	304.9 ± 53.8	53.15 ± 5.60	6.00 ± 0.00	2.88 ± 0.54	1834 ± 225	1859 ± 230
	M	495.2 ± 60.9	19.96 ± 1.30 *	6.00 ± 0.82	3.87 ± 0.58	4654 ± 349 **	4776 ± 322 **
Alphitolic acid	C	526.7 ± 45.6	3.53 ± 0.27	6.67 ± 0.47	3.72 ± 0.46	5446 ± 346	5580 ± 379
	M	171.0 ± 21.9 **	10.25 ± 0.59 **	4.67 ± 0.47	4.18 ± 0.65	1855 ± 126 **	1912 ± 112 **
Maslinic acid	C	899.5 ± 144.4	13.50 ± 0.67	6.00 ± 0.00	2.17 ± 0.36	5026 ± 245	5040 ± 239
	M	578.7 ± 78.6	11.96 ± 1.24	6.00 ± 0.82	3.06 ± 0.13	5879 ± 702	5931 ± 715
Betulinic acid	C	189.5 ± 20.5	33.90 ± 3.03	8.00 ± 1.41	3.58 ± 0.57	1951 ± 180	1995 ± 192
	M	69.8 ± 7.8 *	70.09 ± 4.33 **	1.33 ± 0.24 *	9.00 ± 2.20	774 ± 42 *	945 ± 54 *
Betulonic acid	C	522.7 ± 65.6	13.65 ± 0.61	6.00 ± 0.00	3.74 ± 0.92	3939 ± 98	4107 ± 190
	M	133.2 ± 14.8 *	22.80 ± 0.78 **	3.33 ± 0.47 *	10.49 ± 1.56	1928 ± 205 **	2450 ± 88 **

* *p* < 0.05, ** *p* < 0.01 vs. control group.
